# Supplementary material for: Thermodynamic and structural anomalies of water nanodroplets
Source: Nat Commun. 2018 Jun 19;9:2402. doi: 10.1038/s41467-018-04816-2 (PMC6008328; doi:10.1038/s41467-018-04816-2)
Supplement: Supplementary file 1 — Supplementary Information [file 41467_2018_4816_MOESM1_ESM.pdf]

Supplementary information for:
Thermodynamic and structural anomalies of water nanodroplets

Shahrazad M.A. Malek,[1] Peter H. Poole,[2] and Ivan Saika-Voivod[1]

[1]*Department of Physics and Physical Oceanography, Memorial University of Newfoundland, St. John's, NL, A1B 3X7, Canada*

[2]*Department of Physics, St. Francis Xavier University, Antigonish, NS, B2G 2W5, Canada*

(Dated: May 14, 2018)

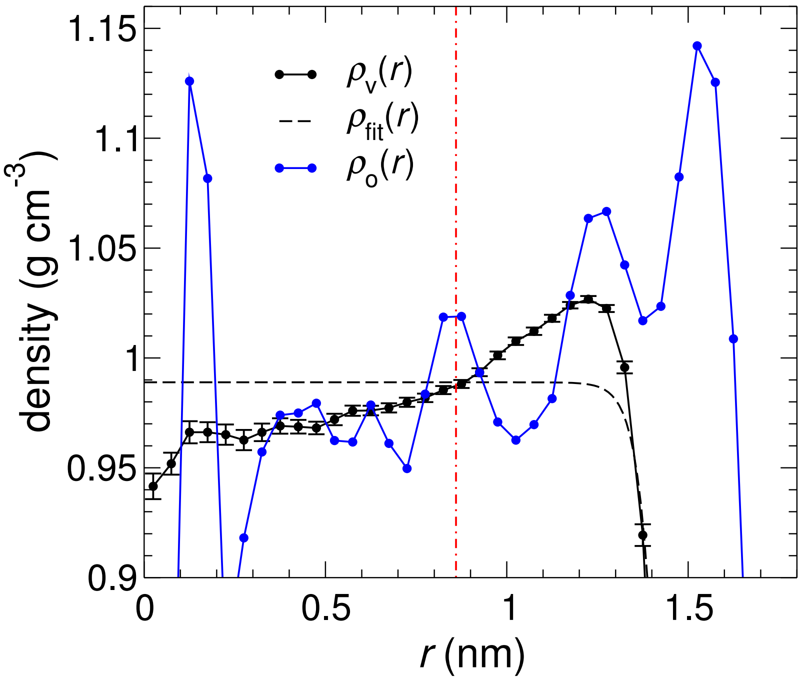

Supplementary Figure 1. Comparison of density profile definitions. Here we plot $\rho_o(r)$ and $\rho_v(r)$ for water nanodroplets with $N = 776$ at $T = 180$ K. The black dashed line is a fit to $\rho_v(r)$ using Eq. 3. The surface region of the droplet, as defined in Methods, is the region where r is larger than that of the red dot-dashed line. Error bars represent one standard deviation of the mean.

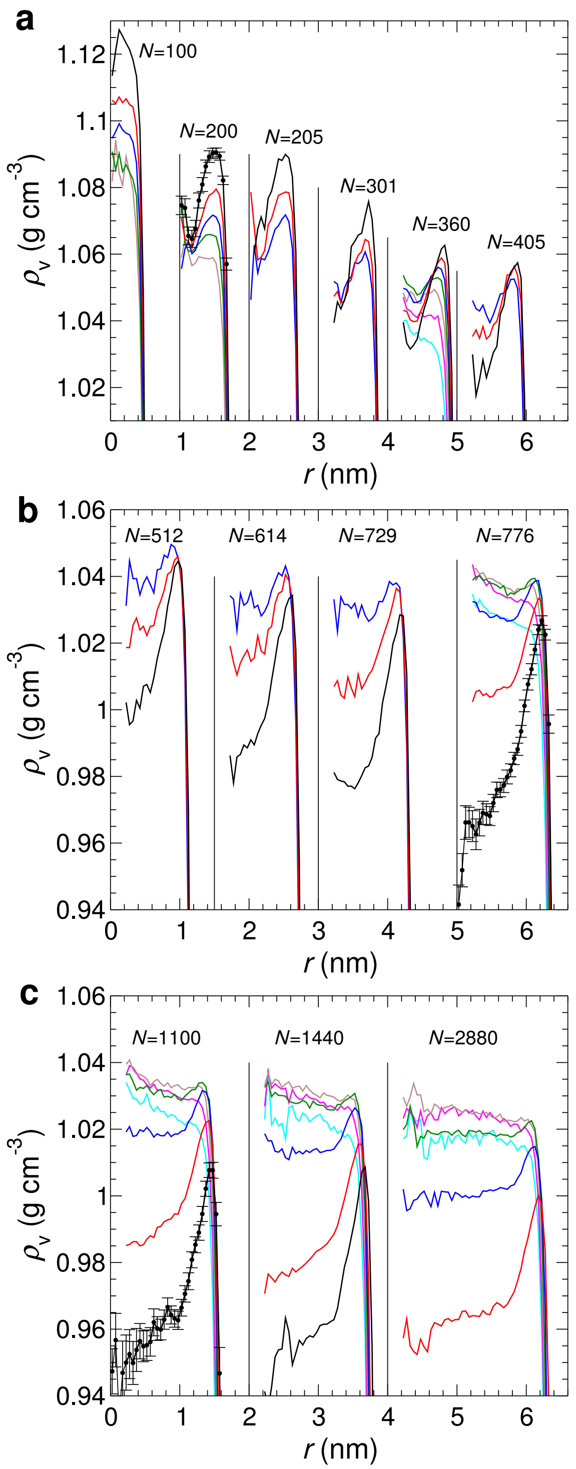

Supplementary Figure 2. Nanodroplet density profiles. Here we show $\rho_{\rm v}(r)$ for a wide range of N and T. In each panel, one representative curve is shown with error bars. For curves without error bars, data for $r < 0.2$ nm are not plotted for $N \geq 301$, since the error at small r is typically large. Line colours indicate T: 180 K (black), 200 K (red), 220 K (blue), 240 K (green), 260 K (brown), 280 K (magenta), 300 K (cyan). To facilitate comparison, for most data sets the origin of r has been shifted by an integer multiple of 0.5 nm, as indicated by the thin vertical lines. Error bars represent one standard deviation of the mean.

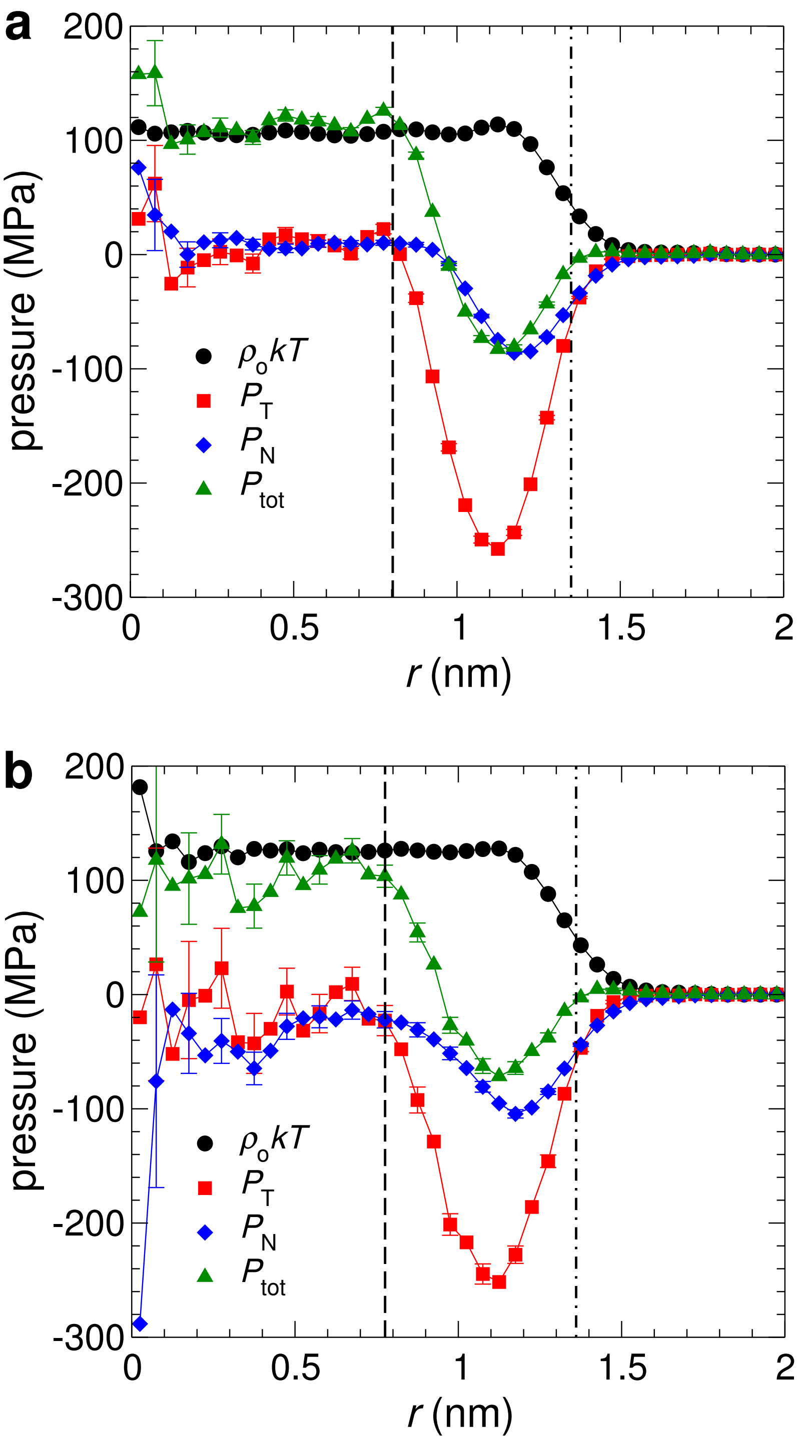

Supplementary Figure 3. Contributions to the pressure inside water nanodroplets. **a** $N = 360$ and $T = 220$ K. **b** $N = 360$ and $T = 260$ K. Vertical lines identify $r = R_L$ (dashed) and $r = R$ (dot-dashed). Error bars represent one standard deviation of the mean.

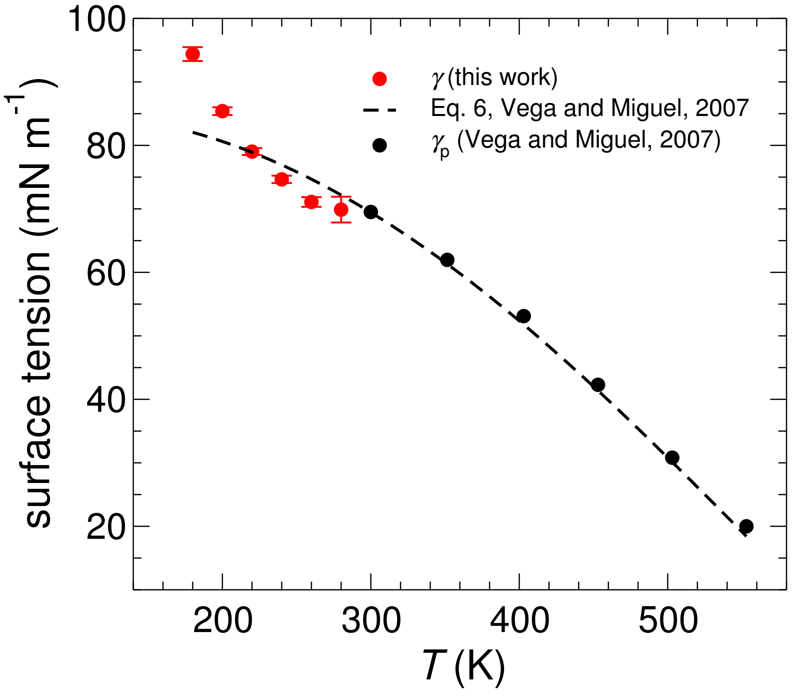

Supplementary Figure 4. Variation of the nanodroplet surface tension with temperature. We compare our results for γ with results for the surface tension γ_{p} of a planar liquid-vapour interface for TIP4P/2005, taken from Ref. 31. Error bars represent one standard deviation of the mean.

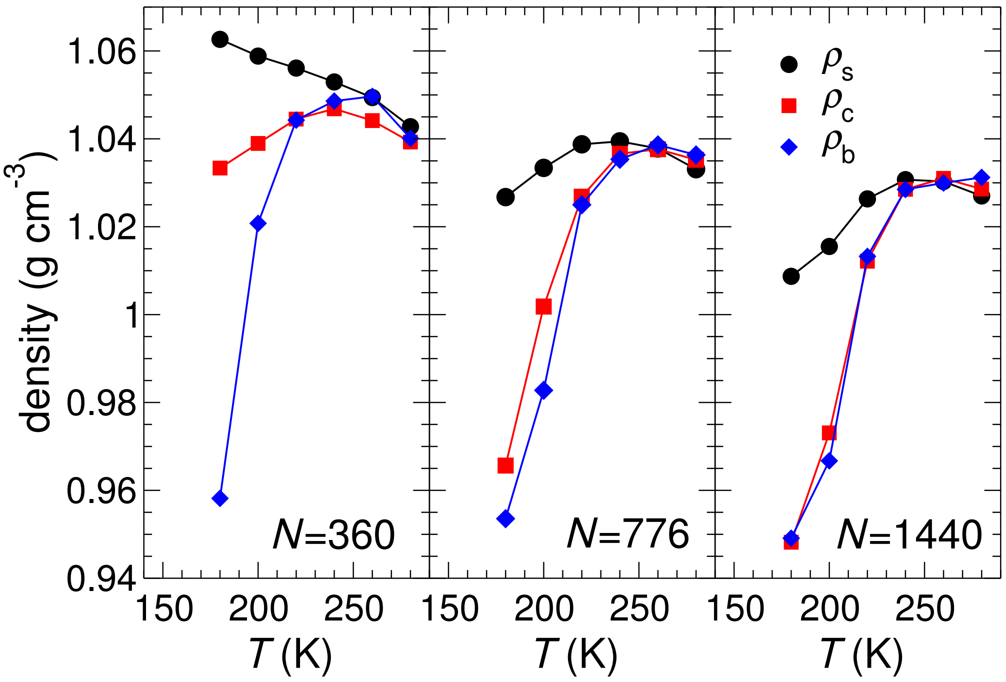

Supplementary Figure 5. Variation with temperature of characteristic densities for nanodroplets. Here we show the dependence of ρ_s, ρ_c and ρ_b on T for droplets of various sizes N. For $N = 1440$, the droplet is large enough for the core density ρ_c to reach the bulk density ρ_b, despite the growing difference between ρ_b and the surface density ρ_s at low T. For smaller droplets, ρ_c does not reach ρ_b at low T.

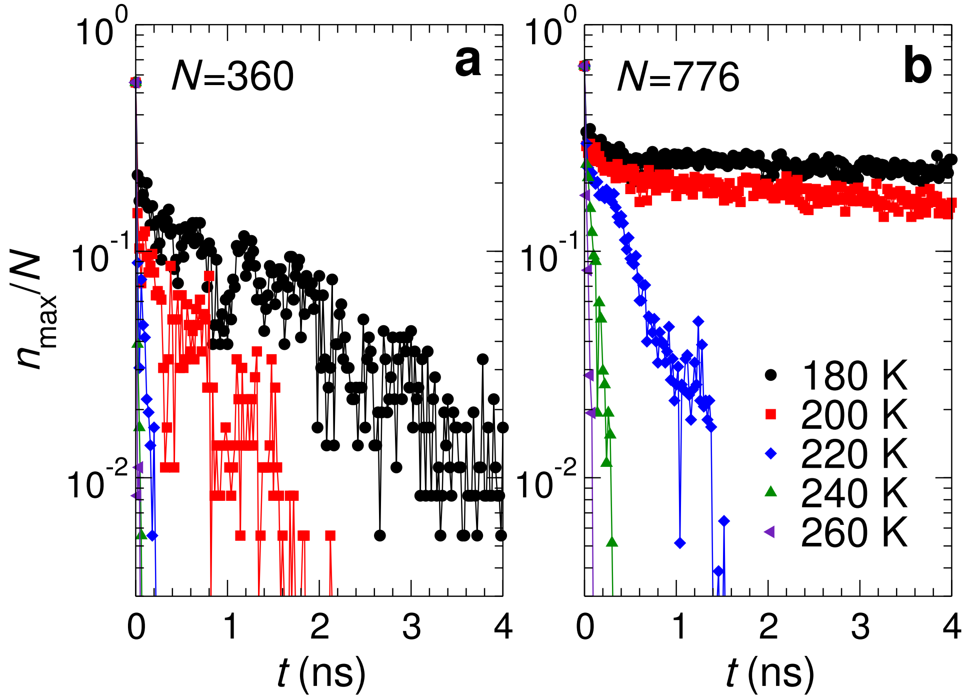

Supplementary Figure 6. Melting of nanocrystals. Variation of $n_{\max}/N$ with time t during melting of ice nanocrystals of size N at various T.

N	L (nm)	T (K)	N_d	τ (ns)	N_τ
100	10	180	100.00	2.4	1167
		200	100.00	0.4	3499
		220	100.00	0.8	2892
		240	99.97	0.8	2995
		260	99.75	0.8	3499
200	10	180	200.00	3.2	864
		200	200.00	0.8	3335
		220	200.00	0.8	3453
		240	199.97	0.8	3452
		260	199.82	0.8	3500
360	10	180	360.00	2.4	392
		200	360.00	0.4	5921
		220	360.00	0.2	12113
		240	360.00	0.2	9837
		260	359.94	0.2	1397
		280	359.71	0.2	876
		290	359.56	0.2	10287
		300	359.26	0.2	10247
776	15	180	776.00	6.8	166
		200	776.00	0.8	954
		220	776.00	0.4	1917
		240	775.87	0.4	1808
		260	775.84	0.4	1921
		280	775.32	0.4	1977
		290	774.84	0.4	2040
		300	773.85	0.4	2009

Supplementary Table I. Run parameters and relaxation time scales for our SLR nanodroplet simulations. Symbols are as defined in the Methods section. For each N, as T increases, τ becomes equal to the time interval between successive stored configurations during each SLR. Since we cannot measure values of τ smaller than this time interval, such a τ value represents an upper bound on the actual value of τ.

N	L (nm)	T (K)	N_d	τ (ns)	N_τ
1100	20	180	1100.00	12.8	46
		200	1100.00	1.6	288
		220	1100.00	0.8	562
		240	1099.75	0.8	577
		260	1099.53	0.8	603
		280	1098.32	0.8	594
		290	1097.01	0.8	556
		300	1095.13	0.8	579
1440	20	180	1440.00	8.6	49
		200	1440.00	1.0	360
		220	1439.98	0.2	1588
		240	1439.77	0.2	1502
		260	1439.74	0.2	1153
		280	1438.64	0.2	829
		290	1437.84	0.2	261
		300	1434.91	0.2	205
2880	20	200	2880.00	1.4	74
		220	2880.00	0.2	490
		240	2879.97	0.2	464
		260	2879.89	0.2	443
		280	2879.44	0.2	240
		290	2878.71	0.2	320
		300	2877.31	0.2	148

Supplementary Table II. Run parameters and relaxation time scales for our SLR nanodroplet simulations. Symbols are as defined in the Methods section. For each N, as T increases, τ becomes equal to the time interval between successive stored configurations during each SLR. Since we cannot measure values of τ smaller than this time interval, such a τ value represents an upper bound on the actual value of τ.

N	L (nm)	T (K)	N_d	τ_s (ns)	M	t_run (ns)
205	8.29	180	205.00	1.63	1000	79.70
		200	205.00	0.14	1000	15.94
		220	205.00	0.05	1000	15.94
301	9.42	180	301.00	1.02	250	15.80
		200	301.00	0.12	250	7.90
		220	301.00	0.04	1000	7.90
405	10.40	180	405.00	1.91	250	63.13
		200	405.00	0.13	1000	7.89
		220	405.00	0.02	1000	7.89
512	11.25	180	512.00	2.36	250	47.71
		200	512.00	0.19	250	7.95
		220	512.00	0.04	250	7.95
614	11.95	180	614.00	2.55	250	96.73
		200	614.00	0.23	250	8.06
		220	614.00	0.05	250	8.06
729	12.65	180	729.00	4.19	250	99.63
		200	729.00	0.28	250	8.27
		220	729.00	0.06	250	8.27

Supplementary Table III. Run parameters and relaxation time scales for nanodroplet simulations carried out using the swarm relaxation method. Symbols are as defined in the Methods section.
